# Vibration Foam Rolling Treatment Influence on Acute Changes in Plantar Flexors Muscle Temperature and Surface Emg Activity in Amateur Male Athletes

**DOI:** 10.3390/jfmk10010025

**Published:** 2025-01-08

**Authors:** Aleksandar Borisavljević, Marko Ćosić, Goran Janković, Iva Radić, Dunja Janković, Milivoj Dopsaj

**Affiliations:** 1 Sports Club Athletic Body Response, 11000 Belgrade, Serbia; 2 Faculty of Sport and Physical Education, University of Belgrade, 11000 Belgrade, Serbiamilivoj.dopsaj@fsfv.bg.ac.rs (M.D.); 3 Institute for Medical Research, University of Belgarde, 11000 Belgrade, Serbia; 4 Faculty of Sport and Psychology, Tims, University of Novi Sad, 21000 Novi Sad, Serbia

**Keywords:** self-myofascial release treatment, foam rolling, maximum voluntary isometric contraction, vibration muscle treatment

## Abstract

**Background/Objectives:** Foam rolling is widespread and deeply rooted in exercise practice. The optimal duration and role of this treatment still lack scientific consensus. A relatively novel foam rolling treatment that combines vibration during application targets different muscle characteristics that are not well understood. Studies exploring this combined treatment are scarce. The aim of this study was set to investigate the acute effects of different duration vibration (15 s, 30 s, and 60 s) foam rolling treatment (VFRt) on muscle skin temperature and surface muscle electromyography (_s_EMG) during Maximal Voluntary Isometric Contraction. **Methods:** Eighteen male subjects performed four sets of three trials of maximal isometric heel rises during three experimental sessions conducted in research laboratory. **Results:** Repeated measures of ANOVA determined that the muscle skin temperature significant difference was only found for the 30 s treatment (*p* = 0.013–0.000). For surface muscle electromyography a 30 s treatment out of all three yielded the most significant results, between pretreatment set and post-treatment set 1 (*p* = 0.01)—small effect size (Cohen’s d = −0.33)—and pretreatment set to post-treatment set 3 (*p* = 0.01)—small effect size (Cohen’s d = −0.30). **Conclusions:** All treatments did not produce significant differences during Maximal Voluntary Isometric Contraction heel rises, which—for practical application purposes—present a safe treatment. Future studies should investigate the acute effects of longer duration treatment on changes in surface muscle temperature. In terms of practical application, other findings suggest that muscle efficiency was improved taken into account of unchanged muscle strength along with decreased _s_EMG, which is beneficial. Also, the downward trend of muscle activity caused by the other two treatment durations could be of significance for practical application during rehabilitation process or during activities where this is a desired and indicated goal. In terms of targeting elevated muscle activity, 30 s of VFRt is the treatment of choice.

## 1. Introduction

Foam rolling has been gaining more research focus in recent years, and still there is a lack of guidelines in terms of optimal duration and the role of this treatment. Physiotherapists and other practitioners commonly prescribe foam rolling as an intervention, but the mechanistic effects of this intervention are not known [1]. Widespread application of this treatment far outweighs the evidence found in the current literature regarding proper and beneficial use. The individual applies their own bodyweight; thus, the applied direct mechanical pressure to the targeted soft tissue is performed by rolling the desired body part over the foam roller. In the literature, this is addressed as self-massage (self-myofascial release). In many exercise-induced processes, foam rolling treatment (FRt) plays several roles, such as improving athletic performance, reducing muscle–tendon–fasciae pain and inflammation, improving muscle mobility by changing the range of motion, etc. [2]. Other previous research has indicated that voluntary activation can be affected by applying mechanical pressure on a muscle tissue [3]. Since many research ventures have focused on cause and effect only, while very few did discuss the potential mechanisms underlying this treatment and propose thixotropy, the exact mechanism still remain unknown. Exercise protocols therefore, such as athletic performance improvement or recovery, may be affected by the muscle property changes caused by the FRt.

**Figure 1:**
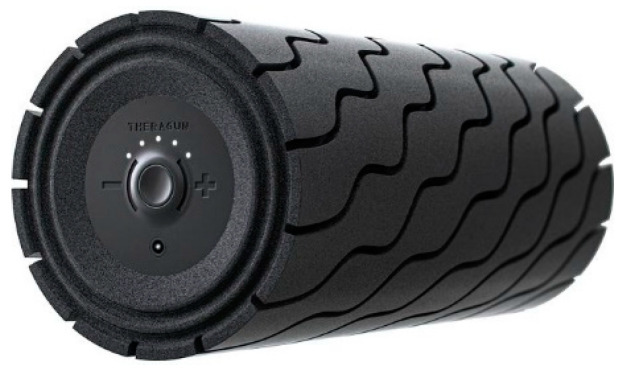
Theragun Wave roller.

As a recent and novel development, a foam roller with vibration (vibration foam roller treatment: VFRt) has been derived, where a battery powered electrical motor inside of a foam roller creates vibrations, with a range of frequency selection that presents a new prop in exercise practice (Figure 1).

Outlining a clear difference between a foam rolling treatment and adding vibration to it tends to provide quite a challenge, since combining two treatments (i.e., rolling and vibration) in the form of a VFRt is creating a gap between the practice and clear scientific evidence in favor of this treatment that has yet to be established. The potential beneficial role of vibration in this treatment could stem from the well-studied effects of vibration training/therapy. WBV (whole-body vibration) studies involving the increase of muscle strength found that it is possible [4]; therefore, adding this type of treatment to an already widespread use of the foam roller is expected to enhance performance. Taking into account that the VFRt provides a range of frequency selection, one should target the range most likely to affect muscle. The motor unit excitation during maximal voluntary contraction frequency is 30 Hz [5]; also, the optimal frequent range is from 30 Hz to 50 Hz to elicit most effective muscle activation, and during vibration training, there are greater improvements in muscle strength and power found for elite athletes then for lower-level athletes [6]. Proper placing of the VFRt in terms of when and where in a single training session or during competition as a pre-warmup, warm-up, or pre-competition treatment is an important question. If misplacing this treatment might have a significant impact on muscle performance that would be needed or desired in the main part of training or during high-intensity or competitive activity, a clearer understanding of the VFRt is obvious. A recent study found that a 30 s FRt and VFRt effectively increased the range of motion and pain pressure threshold, as well as decreased tissue hardness, but the results suggested that there was no advantage using the VFRt over the FRt [7]. In contrast, another study found that the VFRt impacted the multidirectional repeated sprinting-induced muscle damage marker in a protective way, and the authors suggest using the VFRt as a warm-up activity as part of integration into a sport-specific warm-up protocol for elite athletes [8]. Although done on patients, a study using conventional physical therapy with VFRt on a sample of 42 patients from 40 to 60 years in age and of both genders found improvements in hamstring flexibility, pain intensity level, and functional disability of the knee joint [9].

Up to now, studies examining the effects of VFRt are scarce, which is one of the main reasons for proper implementation in exercise practice. The is the first study to our knowledge regarding muscle temperature change measured on a sample of twelve adolescent male squash players utilizing 60 s FRt exercises that found no significant changes in quadricep skin muscle temperature [1]. If thixotropy is one of the possible mechanisms through which acute effects are expressed during FRt or VFRt use, muscle temperature changes should be detectable and examined. Temperature changes could affect the muscle’s contractile properties such as contraction time and force production [10]. Endothelial function was examined in a study on a sample of a study conducted on ten healthy young adults who performed SMR (FRt) with a foam roller in control trials, in a random control crossover design, in the order of adductors, hamstrings, quadriceps, iliotibial band, and trapezius for a duration of 20 repetitions per muscle group, where the plasma nitric oxide (NO) concentration was measured before and 30 min after both SMR and CON trials; the authors of the study found that the plasma NO concentration significantly increased (from 20.4 ± 6.9 to 34.4 ± 17.2 µmol·L^−1^) after SMR using a foam roller (both *p* < 0.05) and proposed that external compression might be a major pathway of vasodilation induced by the increased release of NO [11]. A study on a sample of 20 healthy adult subjects performing isometric squat on a vibrating Galileo platform at a frequency of 26 Hz examined the blood volume in calf muscles and quadriceps muscles by means of a Doppler ultrasound imaging and found significant blood volume increases in the analyzed muscles [12]. Recent study utilizing two FRt exercises (two sets of one minute and two sets of three minutes) implemented to the right anterior thigh of twenty healthy subjects found that local blood flow increased significantly from pre- to post-test (*p* = 0.013), being higher (∆ + 9.7%) in the long-FRt condition, wherein longer FRt durations seem to contribute to better perfusion, which is of interest for exercise professionals and all other exercise experts engaged in designing warm-up and cool-down regimes [13]. Another recent study conducted on 60 subjects found that proprioceptive neuromuscular facilitation stretching (PNFS) done after FRt or VFRt exercises does not provide any additional benefit in improving hamstring flexibility and thigh skin temperatures in comparison to PNFS alone [14]. Since the time and energy of an individual as one of the main resources should be focused on beneficial procedures, it becomes clear that the use of the FRt needs to be addressed if the aim is to improve any type of prewarm-up or warm-up procedure.

Fourteen recreationally trained subjects were tested on two separate occasions in a randomized cross-over design to compare the effects of SS and self-massage (SM) with a roller massage for three sets of 30 s of the calf muscles on electromyography (EMG of the soleus and tibialis anterior) characteristics [15]; the authors found that the EMG values were not affected by either intervention. A study with the aim to determine the effects of applying a roller massager for 20 s and 60 s to the quadriceps muscles on knee joint ROM and dynamic muscular performance on a sample of ten recreationally active men found that 20 s to 60 s of roller massage improved the ROM and muscular efficiency (reduced VL—vastus lateralis—according to the EMG) during a lunge [16]. A recent study involved 21 male subjects who visited the laboratory on two separate days and were randomly assigned to either a vibration foam rolling group (30 Hz) or a non-vibration foam rolling group; both interventions were performed for 3 × 1 min each. Surface electromyography results were analyzed and showed no significant effects on the EMG values during MVIC [17].

So far, researchers have used FRt in the range from five seconds to three minutes, while other implemented sets of FRt, wherein the effect of duration is not well studied [18]. Since thigh muscles seem to contribute marginally to the control of standing balance in healthy, young subjects [19], the role of plantar flexor muscles is important in many activities; therefore, they are set as a focus of interest in this study. When observed in exercise practice, FRt is being administered up to 60 s. Also, analysis of published the literature is showing that, most commonly, FRt duration is up to one minute per muscle group. In terms of its ecological validity and with the aim of this study, this measurement was set to determine the presence of acute effects of different durations of VFRt in the range up to 60 s. The objectives of the study were to uncover the nature of the changes on surface muscle skin temperature (_s_MT) and surface muscle electromyography (_s_EMG) during MVIC (F_max_). Also, the relationship between _s_EMG and MVIC as a measurement of efficiency will be investigated as another objective. From a methodological standpoint, the general hypothesis was that VFRt would not induce any significant changes in the examined properties. The alternative hypothesis was that some of the applied treatment would induce acute changes, i.e., reduce the _s_MT and _s_EMG values.

## 2. Materials and Methods

### 2.1. Experimental Approach to the Problem

**Figure 2:**
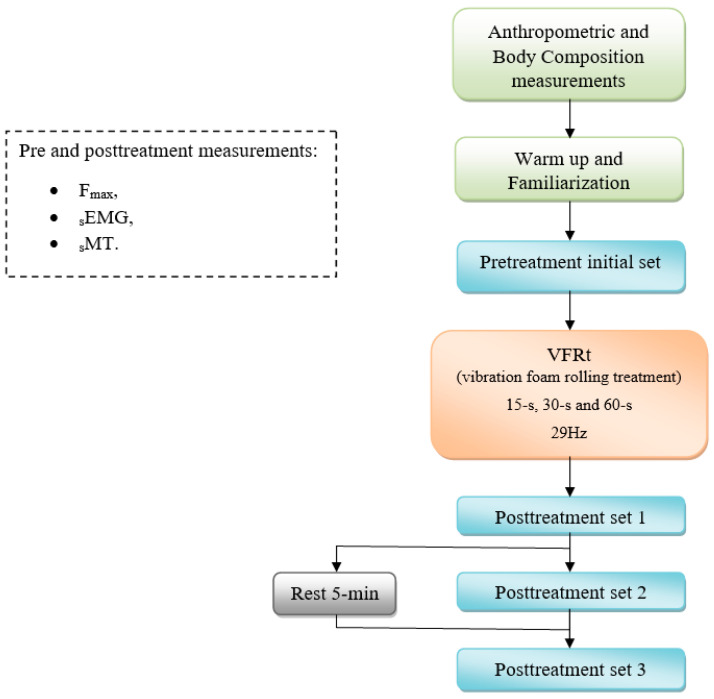
Flow chart of the study design.

A randomized cross-sectional study design was used in a research laboratory setting. Three experimental sessions were scheduled for all subjects at the research laboratory. A priori power analysis for an effect size of 0.5, level of significance *p* < 0.05, power value of 0.80, and one group with 3 measurements generated a required sample size of 9 participants. In accordance with previously published procedures [20], anthropometrics and body composition measurements were taken using an anthropometer and InBody 720 body composition analyzer (Biospace, Seoul, Republic of Korea) during the first session. Figure 2 shows the flowchart of this randomized cross-sectional study design.

Subjects performed standardized general and specific warm-ups. Four sets of three trials of maximal isometric heel rises were done afterwards. As a baseline, the first set of trials was used and labeled as Pretreatment Initial Set (PreTset), while the other three sets were performed after 1, 5, and 10 min of rest, labeled Post-treatment set 1, 2, and 3 (PostTset 1, 2, and 3). Each subject performed three different durations of VFRt: 15 s labeled 15 s VFRt-15-s, 30 s VFRt-30-s, and 60 s VFRt-60-s during experimental sessions, which were done in a random order. All experimental sessions were applied at the same time of the day and were separated by one week. Ambient temperature in the research laboratory was controlled with AC units. During every experimental session, three supervisors were present.

### 2.2. Subjects

Eighteen male adult amateur athletes (competing in lower leagues and up to five training sessions per week of duration up to 90 min) from different sports volunteered to participate in this study. The main inclusion criteria were that they were familiar with foam rolling. Any history of neural conditions and major muscular and/or tendon injury, as well as the use of any medicine during the last month that could affect the outcome of the findings, were the exclusion criteria. The main characteristics of the sample were age = 25.1 ± 4 years, height = 185.1 ± 6 cm, weight = 80.6 ± 7 kg, percent of body fat = 11.2 ± 3.9%, and percent of skeletal muscle mass = 50.8 ± 2.4%. All subjects were instructed to refrain from performing any exercise at least 48 h prior to experimental testing session. Ethics board of the Faculty of Sport and Physical Education, University of Belgrade, under the number III47015 approved the testing and treatment procedures. The study was conducted according to guidelines of the Helsinki Declaration [21].

### 2.3. Procedures

Stationary bicycle lasting five minutes at 70 watts served as a general warm-up protocol. Individually adjusted sitting height was done to achieve full leg extension during cycling and to standardize the warm-up protocol. Specific warm-up consisted of subjects performing standing heel raises (10 repetitions) with focus on fast concentric phase, which was followed by a set of countermovement (10 repetitions) and a set of quick ankle jumps (10–15 repetitions). Each subject had rest for three minutes after specific warm-up during which each was positioned in a testing position and performed three submaximal attempts to get familiar with the setting and instructions. The aforementioned applied warm-up protocol was the same as in the previously published study [22].

### 2.4. Electromyography and Temperature Outputs from MVIC Heel Rise

**Figure 3:**
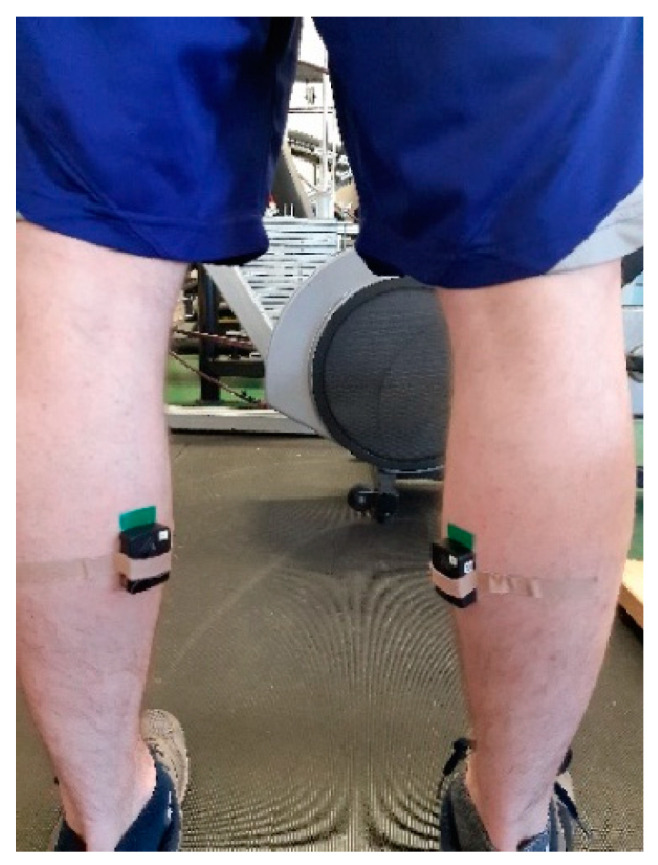
Surface EMG electrodes placement.

According to previously published studies, a standardized test procedure and equipment were used to asses plantar flexor muscles [22,23]. Fixed force transducer on a custom-made construction (tensile/compressive sensitivity 2 mV/n, Hottinger, Type S9, Darmstadt, Germany) for data collection was used, while the acquisition/procession of the signal was performed with software–hardware system (Isometrics Lite, ver. 3.1.1, Sport medical solutions, Belgrade, Serbia). Force transducer and system calibration were tested before each experimental session using a 20 kg weight plate. The activity of the aforementioned muscle of both legs was measured on a premarked, preshaved, and precleaned (Figure 3) spot using an _s_EMG wireless system (Trigno, Delsys, Boston, MA, USA) according to established procedure [24]. Sampling rate was 1925.926; filtering parameters were (window size 0.125 s and window overlap 0.065 s). Clinically validated infrared thermometer (Microlife NC150, Microlife AG, Widnau, Switzerland) was used to measure _s_MT on a premarked spot on the calf muscle of both legs (gastrocnemius muscle medial head). Precision of measurement as a method for instrument calibration was tested before each experimental session against an electrically heated element with a digital display for value (chosen temperature) presentation. The medial head was chosen as the most pronounced spot on the calf for proper sensor placing. ICC values as reliability criteria from trial-to-trial that were checked for the PreTset and calculated for _s_EMG and _s_MT variables (before general warm-up) were 0.894 (95% confidence interval 0.738–0.963) and 0.735 (95% confidence interval 0.452–0.886). Calculated ICC value for _s_MT (after general and specific warm-up and before PreTset) was 0.688 (95% confidence interval 0.328–0.867). ICC value for MVIC during heel rises was 0.819 (95% confidence interval 0.602–0.927).

**Figure 4:**
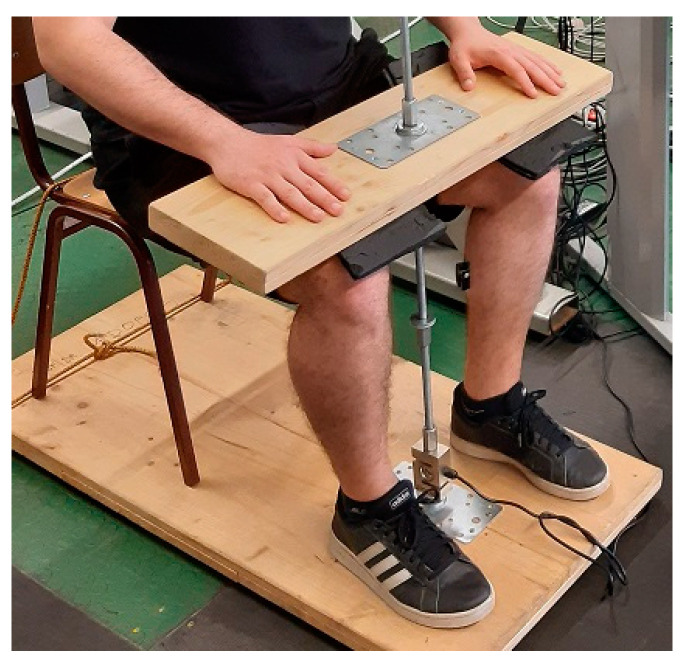
The assessment of outputs during maximal voluntary isometric contraction.

Figure 4 shows a subject in a seated position with hips and knees at 90° of flexion. Subject kept the back straight without leaning on the backrest during trials and with full feet on the floor. A wooden horizontal plate connected to the floor was placed over the thighs. Perpendicularly positioned force transducer was connected with the wooden plate, and the plate was individually adjusted to firmly keep the feet on the ground to prevent the possibility of a heel raise. On the researcher’s word “Go”, the subject had to react and initiate the calf rise as strong and fast as possible. The best of three trials in each set was used for the analysis, while the rest period between trials lasted two minutes. The same testing protocol was repeated immediately after VFRt, after 5 min, and after 10 min rest.

### 2.5. VFRt Protocol

After performing the PreTset, subjects had a 15 min rest period prior to VFRt. A Wave roller (Thera Body, Los Angeles, CA, USA), 30 × 13 cm (Figure 1), was used for the VFRt. The vibration frequency was set to 29 Hz from the range of 5 preset frequencies according to the findings presented in introduction regarding optimal motor unit recruitment. With legs extended and from a seated position on the floor, subjects were using their hands and their bodyweight to press the calves over the roller. Each subject was instructed not to use excessive force so that he felt uncomfortable. One calf was placed over the foam roller, while the other leg was crossed over the leg that rolls. Each subject performed the same number of rolls (2 rolls per second) with the help of a metronome. VFRt was self-administered for 15 s, 30 s, and 60 s over subjects’ soleus and gastrocnemii muscles. In the direction from proximal to distal portion of the calf (muscle belly and tendons), VFRt was executed, which was followed by switching legs and rolling again for the same duration.

### 2.6. Variables

During experimental sessions, three variables were of interest for examination: F_max_, _s_MT, and _s_EMG. The F_max_ was expressed in Newtons (N), _s_MT in degree Celsius (°C), and _s_EMG as RMS (root mean square) in microvolts (µV). For _s_MT and _s_EMG, we obtained values of both legs calculated as an average. Data from _s_EMG were collected during each MVIC trial. Collected _s_MT data were obtained during different time points: before general warm-up (_s_MT-bfr-GW), immediately after general and specific warm-ups (_s_MT-aftr-GSW), immediately after PreTset (_s_MT-PreTset-1 min), after 15 min of rest (_s_MT-PreTset-15 min), immediately after PostTset1 (_s_MT-PostTset1-1 min), and after 5 min of rest (_s_MT-PostTset1-5 min).

### 2.7. Statistical Analysis

The normality of data distribution prior to statistical analyses of VFRt effects was assessed with Shapiro–Wilk test, and all the examined variables showed normal data distribution: F_max_ (*p* = 0.081–0.951), _s_MT-AVG (*p* = 0.054–0.073), and _s_EMG-AVG (*p* = 0.132–0.181). Since the normal distribution criteria were met, parametric statistic tests were used. Also, certain basic descriptive statistics were calculated for all the examined variables such as mean, standard deviation (SD), and coefficient of variation (cV%) as a measure of sample homogeneity. Repeated measures ANOVA with a pairwise comparison that was analyzed by Bonferroni post hoc test was used to analyze the effects of self-administered VFRt. To emphasize the observed differences between and within treatments, a Cohen’s d value was calculated to present effect size. A Cohen’s effect size classification was implemented for result interpretation [25], with the statistical significance set to *p* < 0.05. Correlation among variables was examined using the Pearson coefficient. For significant difference analysis between the found correlations, a Fisher r-to-z transformation was performed. For the subject sample that was available for this research, 18 subjects, a post hoc power analysis was calculated using software G*power version 3.1.9.4., and the generated power value for this sample was 0.76. Statistical analysis was done using the program SPSS for Windows, Release 27.0 (Copyright © SPSS Inc., 1989–2002) (Chicago, IL, USA).

## 3. Results

The descriptive statistics for F_max_ suggest a homogeneous sample according to the cV% values (10.4–16.8), and they are presented in Table 1 along with the repeated-measure ANOVA results. The results in the table show that all three self-administered VFRts did not produce a significant difference between the F_max_ for the PreTsets and PostTsets.

The descriptive statistics for the _s_MT and repeated-measure ANOVA results are presented in Table 2. The cV% values suggest a homogeneous sample (0.8–1.7). The repeated-measure ANOVA determined that there was no significant difference between any of the self-administered VFRts. A significant difference was only found for the VFRt-30-s, and a detail view of the results is presented in the Table 3.

**Figure 5:**
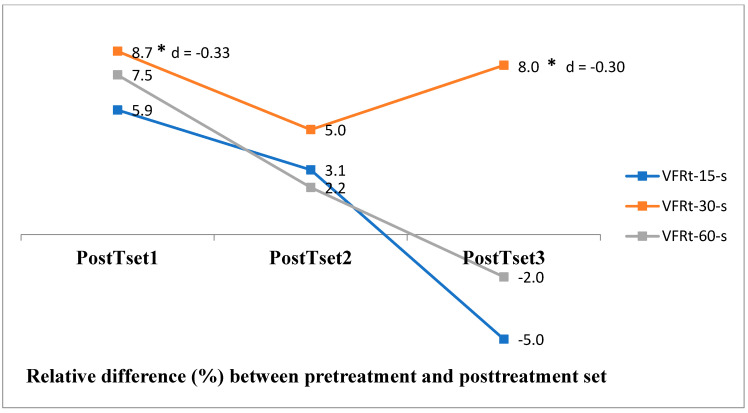
Surface EMG relative difference (%) between pretreatment and post-treatment set of each vibration foam rolling treatments.

The descriptive statistics for the _s_EMG and repeated-measure ANOVA results are presented in Table 4. The cV% values suggest a homogeneous sample (22.6–34.2). A detailed repeated-measure ANOVA analysis found a significant difference between VFRt-15-s and VFRt-60-s on a PostTset2 level—medium effect size (Cohen’s d 0.45)—and between VFRt-30-s and VFRt-60-s on a level PostTset3—medium effect size (Cohen’s d = 0.60). Within VFRt-15-s, a significant difference was found only between PostTset1 and PostTset3 (*p* = 0.02). The VFRt-30-s test out of all three yielded the most significant results: between PreTset and PostTset1 (*p* = 0.01)—small effect size (Cohen’s d = −0.33)—and PreTset to PostTset3 (*p* = 0.01)—small effect size (Cohen’s d = −0.30). Within VFRt-60-s, a significant difference was found between PostTset1 and PostTset3 (*p* = 0.008). The relative difference (%) between the initial testing (PreTset) and PostTset of each VFRt is presented in (Figure 5), significant difference is marked with *. A decreasing trend of the results is visible for VFRt-15-s and VFRt-60-s, although it is not statistically significant. For VFRt-30-s, a positive trend of the results is visible, being statistically significant at the PostTset1 and PostTset3 levels.

**Figure 6:**
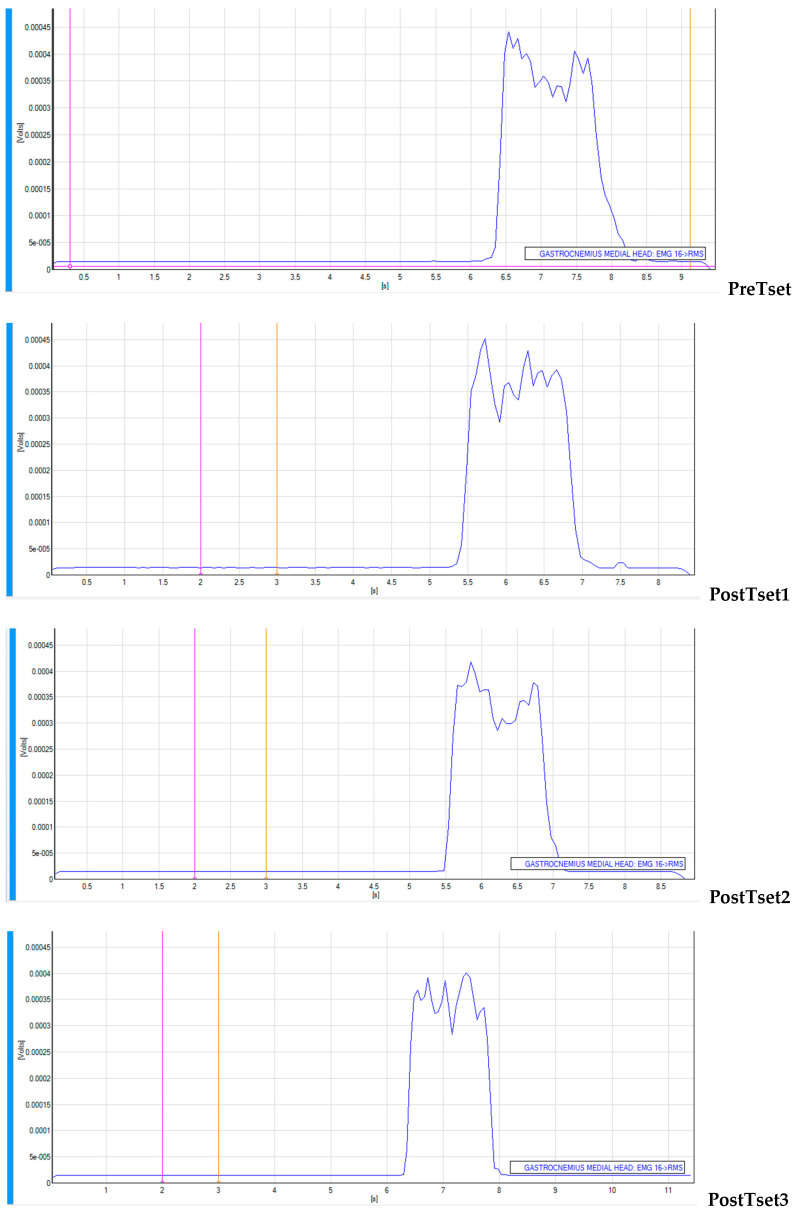
Surface EMG pattern over time for vibration foam rolling treatment 15 s.

**Figure 7:**
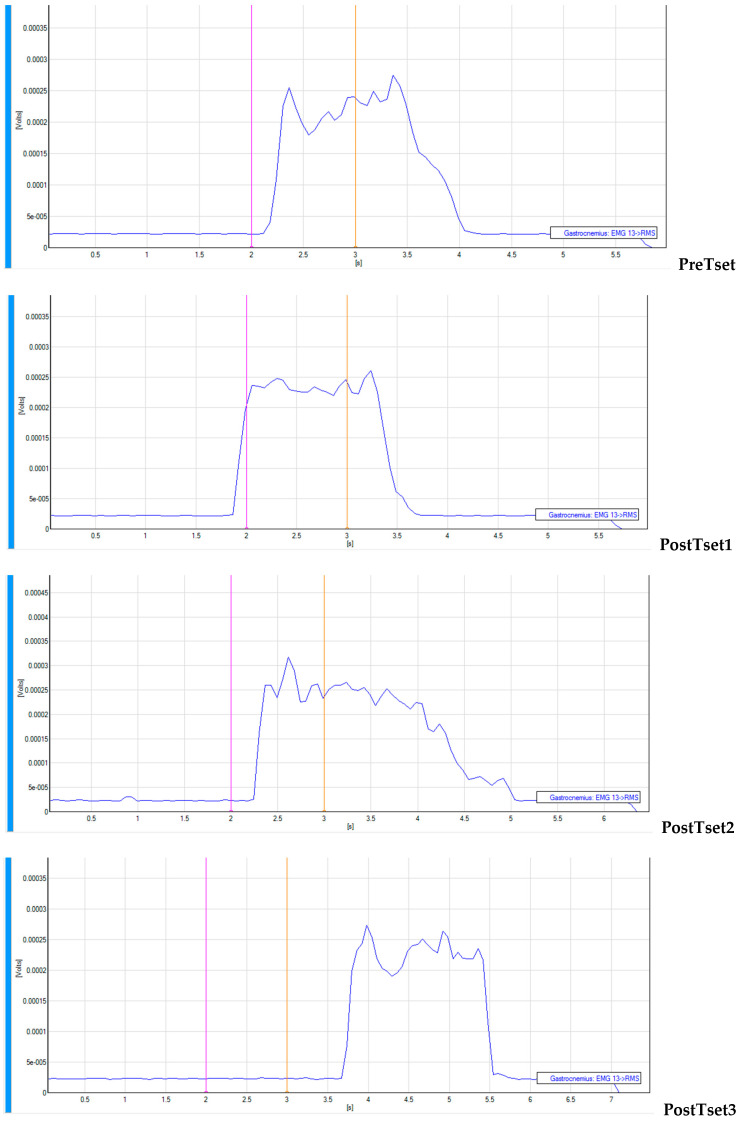
Surface EMG pattern over time for vibration foam rolling treatment 30 s.

**Figure 8:**
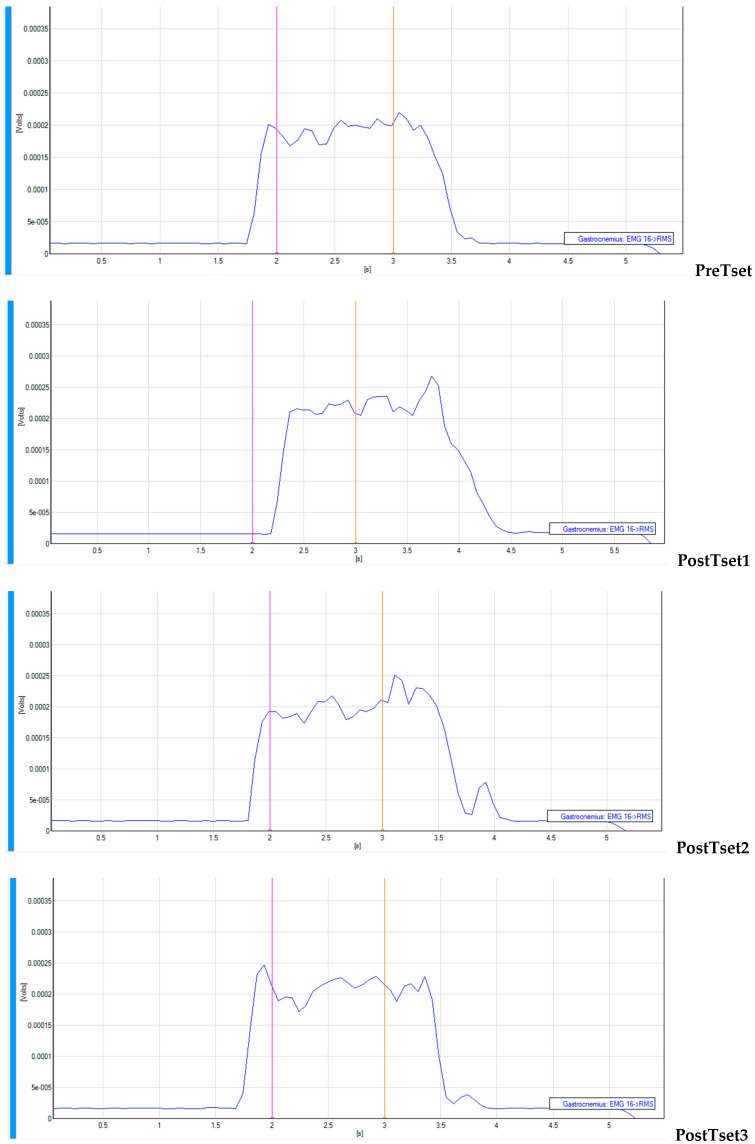
Surface EMG pattern over time for vibration foam rolling treatment 60 s.

Patterns of _s_EMG values over time from a subject are presented in Figure 6 (VFRt-15-s), Figure 7 (VFRt-30-s), and Figure 8 (VFRt-60-s).

The results of the correlation analysis among variables are presented in Table 5. For the pretreatment correlation analysis and for the post-treatment, the significant values are presented in bold.

In regard to the results of correlation analysis presented in Table 5, the only significant result using Fisher r-to-z transformation was found between sEMG_60 s and sMT_15 s in relation from pretreatment to post-treatment, a z value of −1.71, and a *p* value of 0.0436.

## 4. Discussion

The impact of different duration self-administered vibration foam rolling treatment (15 s, 30 s, and 60 s) on the _s_MT and _s_EMG values of plantar flexors during MVIC was set as the aim of our study. The results of our study suggest that none of the VFRts applied here affected the F_max_. Regarding the _s_MT results, these suggest that only VFRt-30-s affected the plantar flexors in a negative way. Also, an interesting finding is that only the VFRt-30-s affected the plantar flexors _s_EMG value, causing a significantly elevated activity. The other two applied VFRts induced a decrease in the _s_EMG but without significance.

In terms of acute effects on F_max_, the results of our study are in agreement with the findings from the study where a higher vibration frequency (48 Hz) and duration was administered through three sets of 60 s for the calf muscles [26,27]. In contrast, a study that implemented a similar isometric testing setup to our study but with the exception of the treatment and testing being done on one leg using the same muscle group as in our study design applied a roller stick self-massage without vibration for a duration of three sets of 30 s that induced a significantly greater force production and a small increase in the MVIC, which was observed ten minutes after treatment [15]. An increase by two fold of quadriceps isokinetic muscle strength was found in a study where VFRts (28 Hz) of durations of three sets of 30 s were administered on hamstring and quadriceps muscles [28], which is contrast with our finding, especially taking into consideration the use of almost the same frequency and duration. A plausible explanation given by the authors of the study where a massage exhibited lower activation and elevated parasympathetic response of the vastus medialis during MVC suggests that it could be attributed to a transient loss of muscle strength or a change in the muscle fiber tension-length relationship [29]. Future research should focus on providing a clear understanding of mechanisms involved in changes of muscle strength properties during and after the implementation of this self-treatment, since many have been proposed but without consensus so far.

Increased muscle contractile capacity occurs with higher muscle temperature after acute bouts of passive heating, and it is also enhanced when voluntary and involuntary fast force contraction properties are apparent [30]. Passive heating suggests that increased muscle temperature is more effective on fast contraction force (e.g., RFD and time to peak torque) and is a probable mechanism responsible for this is the release of Ca^2+^ into the myoplasm, which results in the binding of Ca^2+^ to troponin C unblocking the sites between the actin and myosin heads (cross-bridges formation), subsequently producing force development [30]. Regardless of its implementation in the field of sport or rehabilitation, VFRt is not a passive technique; similar acute effects could be expected and are of importance for exercise practice. The findings of our study are the first to our knowledge to present the acute effects of different durations of VFRts on surface muscle temperature. The findings in our study regarding no change during 60 s treatment are in agreement with the findings of the study of [1]. The other finding in our study that found that the VFRt-30-s exercise produced statistically significant and decreasing results for the _s_MT is somewhat surprising. It was to be expected that longer durations, friction should induce greater changes in the _s_MT. Increases in blood flow, and subsequently in muscle fluid, in response to passive heating may also increase the rate of cross-bridge formation and the muscle shortening velocity in addition to increasing muscle stiffness, causing a positive effect on the RFD [30]. It is to be expected that the same mechanism could be responsible during VFRt. Local muscle blood flow increases linearly with muscle temperature [31] and can rise by 61% after heat exposure [32]. FRt could be one of the interventions among others to prevent the detrimental effects of limb immobilization on skeletal muscle health. Daily exposure to heat stress results in increased heat shock protein expression, maintains mitochondrial respiratory capacity, attenuates atrophy in skeletal muscle, and may serve as an effective therapeutic strategy [33]. While no single therapeutic intervention may offer the physical, physiological, and mental benefits of exercise, exercise mimetic strategies have the potential to provide at least some like benefits, especially for the sedentary and clinical population [34]. Passive heating may offer exercise mimetic hypertrophy and neuromuscular adaptations that could resultantly increase the quality of life and decrease healthcare costs [35]. Future studies should investigate longer duration VFRt and utilize other sensitive devices for detecting muscle temperature changes during and after VFRt.

The main finding of our study regarding changes in _s_EMG is the differentiation of one duration treatment that led to elevated acute effects 20 min after and that was VFRt-30-s. This finding is the first to our knowledge in the published literature to yield such statistically significant results. A plausible explanation could stem from the fact that a short VFRt causes contractions that inhibit adequate motor unit action potential generation via underlying unknown neural mechanism, while the longer duration VFRt could have generated elevated parasympathetic activity that had a relaxing acute effect on the muscles; therefore, a 30 s VFRt may be the optimal duration for elevated _s_EMG activity. Future research should address this issue, since clearly different duration differentiate the desired outcome. Within VFRt-15-s and VFRt-60-s, a significant difference was found between their PostTset1 and PostTset3 results, which could suggest that fatigue did occur, but the findings of VFRt-30-s and no changes in F_max_ suggest otherwise. Even though VFRt-15-s and VFRt-60-s caused an insignificant decrease in relationship to the baseline (pretest) _s_EMG, this finding could be interpreted as improved muscle efficiency, since for the unchanged F_max_ values, the muscles exhibited lower activity after treatment. Such a finding is in agreement with the finding of the study, where foam rolling treatment in duration of 2 min during three consecutive days caused a decrease in _s_EMG during the same submaximal task (50% of MVC), which authors interpretedas improved muscle efficiency [36]. In terms of improved efficiency, the results of this study are in agreement with the findings of [16]. The authors of that study concluded that while performing the same motoric task (lunge), lower EMG activity was recorded for the muscle Vastus lateralis; therefore, the muscle efficiency was improved. The same authors also state that foam rolling is an active process, since during their treatment, the recorded surface EMG activity was 7% and 8% of the MVIC for the muscles Biceps femoris and Vastus lateralis. Contractions are a normal human response to a potential unpleasant or during an unpleasant situation such as FRt [37]. In contrast, a study on 14 subjects during which the effects of static stretching and foam rolling (roller massager) were compared, both treatments for three sets of 30 s, the recorded values of surface EMG activity of the muscles soleus and tibialis anterior showed no significant difference [15]. In agreement with previously mentioned study are the results of the study done on 21 male active subjects (mean age 25.2 ± 3.8) where the effects of VFR (30 Hz) and FR were compared in terms of surface EMG, and the results showed no significant difference between two treatments during MVIC testing [17].

Whole-body vibration in a study where 45 Hz frequency was applied during isometric squat for five sets of 1 min to record the surface EMG revealed that the H-reflex and M-wave amplitude values were decreased comparing to baseline measurement and remained decreased for 20 min after the vibration treatment [38]. On the sample of 11 subjects, during 2 min FRt of quadriceps muscles, the H reflex value was found to return to the baseline values after two minutes of rest after cessation of the treatment, thus allowing for the activation of deep mechanoreceptors to decrease and the normal production of force [39]. In contrast [40], the findings of the study suggest that FRt of plantar flexor muscles did not change the relationship of H/M waves as a measure of spine excitability independent of gender. When interpreting _s_EMG activity, one should approach with caution. A plausible explanation can be found in the study of [41], which offers an interpretation that EMG amplitude is a poor determinant of neural activation. A larger subject sample size could provide more valid findings of this study. Also, research has shown that differences in muscle architecture can influence EMG amplitude, even when the muscle is activated at a similar intensity [42]. For small muscles, the relationship between force and the EMG signal tend to be linear, whereas in bigger muscles that need better motor recruitment, the same relationship tends to not be linear, because the amplitude variations of the muscle electric signal do not correspond to the force variations [43]. In a study with a purpose to evaluate a possible linear relationship between the RMS value of the EMG signal and the contraction force of the rectus femoris, vastus medialis, lateralis, biceps femoris, semitendinosus, and brachial biceps muscles on a sample of 24 female university students that practiced physical activity regularly results showed that a linear relationship with the required torque was found between the contraction force and the RMS value of the EMG signal in females for the analyzed muscles [44]. Even though many correlations have been found according to the results from Table 5, the only significant one that could be attributed to VFRt was between sEMG_60 s and sMT_15 s. In terms of practical application, our subject sample provides certain limitations for the extrapolation of our study findings to other types of population; nevertheless, exercise and health practitioners could take these findings into consideration, especially for those that would benefit, like the elderly population and populations with certain health impairments. Future research should focus on different subject samples, i.e., population and gender, that would benefit from this type of treatment and provide clearer guidelines under which circumstances it is recommendable or not. Since the vibration foam roller used here offers a range of frequency and we implemented only one, this could also present a limitation for this study and a guideline for other future studies. Lastly, when considering the individual variability in terms of the selection of subjects in the first place and response to the applied treatments in the second place, one should take into account as a limitation and a future issue that must be addressed, which is the fact that subjects could vary in muscle stiffness, which reflects to joint flexibility and therefore the outcomes of the treatment wherein the cause for this variability could stem from previous accumulated fatigue, existing deficits, etc.

## 5. Conclusions

The data from our study suggest that short-duration application of up to one minute of self-administered myofascial release treatment with a vibrating foam roller (VFRt) presents a safe technique for plantar flexor muscle strength properties, which is of significance for practical application. Specifically, in terms of maximal muscle force production, that outcome remained unchanged after all of the three administered durations. Application of this short duration treatment did not provide any meaningful detectable surface muscle temperature changes that could be of practical importance at this moment. Our findings regarding surface EMG changes indicate that the use of VFRt-30 s in terms of practical application should be implemented if the desired outcome of this treatment is elevated muscle activity. Although the VFRt-15 s and VFRt-60 s revealed a downward trend of muscle activity, when taken into consideration with the finding of unchanged muscle strength, it can be concluded that muscle efficiency was improved, which is of practical importance in the field of sports, recreation, and, especially, rehabilitation. Also, as a practical application guideline, a downward trend of muscle activity caused by these two treatment durations could be of significance during rehabilitation processes for certain conditions where it is beneficial in terms of health to decrease the muscle activity.

## Figures and Tables

**Table 1:** Descriptive and repeated measure ANOVA indicators for F_max_.

Testing	Experimental Session				
PreTset	F_max__15-s	F_max__30-s	F_max__60-s	Wilks’ Lambda	*p*
Mean ± SD	3569 ± 373	3651 ± 484	3672 ± 620	0.93	0.57
cV%	10.4	13.2	16.8		
95% Confidence Interval for Mean (Lower–upper bound)	3383.37–3757.74	3410.25–3892.53	3364.30–3981.36		
	VFRt-15-s	VFRt-30-s	VFRt-60-s		
PostTset1	3513 ± 366	3585 ± 441	3593 ± 571	0.94	0.61
	10.4	12.3	15.9		
	3331.41–3696.15	3365.84–3805.28	3308.73–3877.27		
PostTset2	3509 ± 421	3560 ± 474	3574 ± 537	0.96	0.74
	12.0	13.3	15.0		
	3299.58–3718.64	3324.91–3796.53	3307.27–3841.73		
PostTset3	3506 ± 368	3607 ± 557	3570 ± 588	0.94	0.63
	10.5	15.4	16.4		
	3323.38–3689.73	3330.37–3884.63	3277.71–3862.63		
Wilks’ Lambda	0.94	0.85	0.84		
*p*	0.82	0.48	0.46		

**Table 2:** Descriptive and repeated measure ANOVA indicators for _s_MT.

Testing	Experimental Session				
_s_MT-bfr-GW	_s_MT_15-s	_s_MT_30-s	_s_MT_60-s	Wilks’ Lambda	*p*
Mean ± SD	35.62 ± 0.32	35.72 ± 0.49	35.81 ± 0.48	0.85	0.24
cV%	0.9	1.4	1.3		
95% Confidence Interval for Mean (Lower–upper bound)	35.47–35.77	35.49–35.95	35.59–36.03		
_s_MT-aftr-GSW	35.42 ± 0.61	35.80 ± 0.42	35.86 ± 0.40	0.48	0.21
	1.7	1.2	1.1		
	35.13–35.70	35.59–35.99	35.66–36.04		
_s_MT-PreTset-1 min	35.82 ± 0.39	35.95 ± 0.22	35.91 ± 0.34	0.87	0.28
	1.1	0.6	0.9		
	35.63–36.00	35.84–36.05	35.75–36.06		
_s_MT-PreTset-15 min	35.71 ± 0.33	35.54 ± 0.43	35.75 ± 0.33	0.82	0.16
	0.9	1.2	0.9		
	35.55–35.85	35.34–35.74	35.59–35.90		
	VFRt-15-s	VFRt-30-s	VFRt-60-s		
_s_MT-PostTset1-1 min	35.65 ± 0.27	35.50 ± 0.42	35.72 ± 0.28	0.78	0.11
	0.8	1.2	0.8		
	35.52–35.77	35.30–35.70	35.58–35.84		
_s_MT-PostTset1-5 min	35.63 ± 0.27	35.51 ± 0.41	35.71 ± 0.28	0.80	0.14
	0.8	1.2	0.8		
	35.50–35.75	35.31–35.69	35.58–35.84		
Wilks’ Lambda	0.52	0.39	0.64		
*p*	0.06	***0.003*** *	0.11		

* The mean difference is significant at the 0.05 level.

**Table 3:** Repeated-measure ANOVA indicators for _s_MT in VFRt-30-s.

Pairwise Comparisons						
_s_MT (°C)		Mean Diff. (I–J)	Std. Error	Sig. ^b^	95% Confidence Interval for Difference ^b^	
					Lower Bound	Upper Bound
_s_MT-bfr-GW	_s_MT-aftr-GSW	−0.08	0.07	1.00	−0.33	0.18
	_s_MT-PreTset-1 min	−0.23	0.10	0.49	−0.56	0.10
	_s_MT-PreTset-15 min	0.18	0.12	1.00	−0.23	0.59
	_s_MT-PostTset1-1 min	0.22	0.09	0.34	−0.08	0.51
	_s_MT-PostTset1-5 min	0.21	0.09	0.33	−0.07	0.50
_s_MT-aftr-GSW	_s_MT-PreTset-1 min	−0.15	0.07	0.67	−0.39	0.09
	_s_MT-PreTset-15 min	0.26	0.11	0.38	−0.10	0.61
	_s_MT-PostTset1-1 min	0.293 *	0.07	***0.013*** *	0.04	0.54
	_s_MT-PostTset1-5 min	0.290 *	0.07	***0.011*** *	0.05	0.53
_s_MT-PreTset-1 min	_s_MT-PreTset-15 min	0.410 *	0.09	***0.004*** *	0.10	0.72
	_s_MT-PostTset1-1 min	0.446 *	0.07	***0.000*** *	0.20	0.69
	_s_MT-PostTset1-5 min	0.443 *	0.07	***0.000*** *	0.21	0.68
_s_MT-PreTset-15 min	_s_MT-PostTset1-1 min	0.04	0.10	1.00	−0.31	0.39
	_s_MT-PostTset1-5 min	0.03	0.10	1.00	−0.32	0.38
_s_MT-PostTset1-1 min	_s_MT-PostTset1-5 min	0.00	0.01	1.00	−0.03	0.02

Based on estimated marginal means. *. The mean difference is significant at the 0.05 level. ^b^. Adjustment for multiple comparisons: Bonferroni.

**Table 4:** Descriptive and repeated-measure ANOVA indicators for _s_EMG.

Testing	Experimental Session				
PreTset	_s_EMG_15-s	_s_EMG_30-s	_s_EMG_60-s	Wilks’ Lambda	*p*
Mean ± SD	276.84 ± 84.74	255.35 ± 69.02	239.80 ± 76.66	0.75	0.18
cV%	30.6	27.0	32.0		
95% Confidence Interval for Mean (Lower–upper bound)	227.91–325.76	215.50–295.21	195.54–284.07		
	VFRt-15-s	VFRt-30-s	VFRt-60-s		
PostTset1	293.13 ± 88.13	277.48 ± 62.79	257.90 ± 81.20	0.67	0.09
	30.1	22.6	31.5		
	242.25–344.02	241.22–313.73	211.01–304.79		
PostTset2	285.29 ± 91.80	268.01 ± 67.37	245.13 ± 83.77	0.52	***0.02*** *
	32.2	25.1	34.2		
	232.28–338.30	229.11–306.91	196.76–293.50		
PostTset3	263.14 ± 88.71	275.79 ± 65.44	234.00 ± 71.89	0.36	***0.002*** *
	33.7	23.7	30.6		
	211.91–314.35	238.00–313.56	193.48–276.50		
Wilks’ Lambda	0.47	0.27	0.42		
*p*	***0.03*** *	***0.002*** *	***0.002*** *		

* The mean difference is significant at the 0.05 level.

**Table 5:** Correlation analysis.

Correlations Among Variables Before and After VFRt										
		_s_EMG_15 s	F_max__15 s	_s_MT_15 s	_s_EMG_30 s	F_max__30 s	_s_MT_30 s	_s_EMG_60 s	F_max__60 s	_s_MT_60 s
_s_EMG_15 s	Pearson Correlation	1	0.161	−0.268	***0.807*** **	0.433	−0.239	***0.621*** **	0.290	−0.097
	Sig. (2-tailed)		0.524	0.282	***0.000***	0.073	0.340	***0.006***	0.243	0.701
	N	18	18	18	18	18	18	18	18	18
F_max__15 s	Pearson Correlation	0.126	1	−0.368	0.217	***0.716*** **	−0.319	***0.570*** *	***0.711*** **	−0.132
	Sig. (2-tailed)	0.618		0.132	0.388	***0.001***	0.196	***0.013***	***0.001***	0.602
	N	18	18	18	18	18	18	18	18	18
_s_MT_15 s	Pearson Correlation	***−0.478*** *	−0.427	1	−0.384	−0.138	***0.789*** **	***−0.484*** *	−0.163	0.462
	Sig. (2-tailed)	***0.045***	0.077		0.116	0.586	***0.000***	***0.042***	0.518	0.053
	N	18	18	18	18	18	18	18	18	18
_s_EMG_30 s	Pearson Correlation	***0.802*** **	0.328	***−0.701*** **	1	0.346	−0.314	***0.761*** **	0.322	0.064
	Sig. (2-tailed)	***0.000***	0.184	***0.001***		0.159	0.204	***0.000***	0.193	0.800
	N	18	18	18	18	18	18	18	18	18
F_max__30 s	Pearson Correlation	0.064	***0.686*** **	−0.304	0.190	1	−0.198	***0.533*** *	***0.731*** **	−0.185
	Sig. (2-tailed)	0.800	***0.002***	0.221	0.451		0.430	***0.023***	***0.001***	0.462
	N	18	18	18	18	18	18	18	18	18
_s_MT_30 s	Pearson Correlation	−0.428	0.099	***0.656*** **	***−0.522*** *	0.029	1	***−0.551*** *	−0.256	0.345
	Sig. (2-tailed)	0.077	0.696	***0.003***	***0.026***	0.910		***0.018***	0.304	0.161
	N	18	18	18	18	18	18	18	18	18
_s_EMG_60 s	Pearson Correlation	***0.745*** **	0.382	***−0.819*** **	***0.866*** **	0.269	***−0.570*** *	1	***0.600*** **	−0.050
	Sig. (2-tailed)	***0.000***	0.118	***0.000***	***0.000***	0.280	***0.013***		***0.009***	0.845
	N	18	18	18	18	18	18	18	18	18
F_max__60 s	Pearson Correlation	−0.157	***0.715*** **	−0.176	0.039	***0.815*** **	0.218	0.111	1	0.222
	Sig. (2-tailed)	0.535	***0.001***	0.485	0.877	***0.000***	0.385	0.662		0.376
	N	18	18	18	18	18	18	18	18	18
_s_MT_60 s	Pearson Correlation	−0.025	−0.206	−0.069	−0.086	−0.434	0.060	0.050	−0.096	1
	Sig. (2-tailed)	0.922	0.412	0.786	0.735	0.072	0.814	0.843	0.706	
	N	18	18	18	18	18	18	18	18	18

** Correlation is significant at the 0.01 level (2-tailed). * Correlation is significant at the 0.05 level (2-tailed). **Important notice:** Underlined values present post-treatment analysis results.

## Data Availability

The original contributions presented in this study are included in the article. Further inquiries can be directed to the corresponding author.
